# Association of large intergenic noncoding RNA expression with disease activity and organ damage in systemic lupus erythematosus

**DOI:** 10.1186/s13075-015-0632-3

**Published:** 2015-05-21

**Authors:** Yanfang Wu, Feifei Zhang, Jianyang Ma, Xiaoyan Zhang, Lingling Wu, Bo Qu, Shiwei Xia, Shunle Chen, Yuanjia Tang, Nan Shen

**Affiliations:** Shanghai Institute of Rheumatology, Department of Rheumatology, Renji Hospital, School of Medicine, Shanghai Jiao Tong University, Shan Dong Middle Road, Shanghai, 200001 People’s Republic of China; Institute of Health Sciences, Shanghai Jiao Tong University School of Medicine (SJTUSM) and Shanghai Institutes for Biological Sciences (SIBS), Chinese Academy of Sciences (CAS), Yue Yang Road, Shanghai, 200031 People’s Republic of China; Division of Rheumatology and the Center for Autoimmune Genomics and Etiology (CAGE), Cincinnati Children’s Hospital Medical Center, 3333 Burnet Avenue, Cincinnati, OH 45229 USA

## Abstract

**Introduction:**

Despite growing evidence that large intergenic noncoding RNAs (lincRNAs) can regulate gene expression and widely take part in normal physiological and disease conditions, our knowledge of systemic lupus erythematosus (SLE)-related lincRNAs remains limited. The aim of this study was to detect the levels of four lincRNAs *(ENST00000500949: linc0949, ENST00000500597: linc0597, ENST00000501992: linc1992,* and *ENST00000523995: linc3995)* involved in innate immunity in the peripheral blood mononuclear cells (PBMCs) of patients with SLE and correlate these lincRNA levels with disease activity, organ damage, clinical features and medical therapies.

**Methods:**

PBMCs were obtained from 102 patients with SLE, 54 patients with rheumatoid arthritis (RA) and 76 healthy donors. lincRNA expression levels were measured by real-time quantitative polymerase chain reaction. Disease activity was assessed using the Systemic Lupus Erythematosus Disease Activity Index 2000 (SLEDAI-2K) scores, and organ damage was evaluated with the Systemic Lupus International Collaborating Clinics/American College of Rheumatology Damage Index.

**Results:**

*linc0949* and *linc0597* were significantly decreased in patients with SLE compared with patients with RA and healthy control subjects. *linc0949* was correlated with SLEDAI-2K score (*r* = −0.329, *P* = 0.0007), as well as with complement component C3 level (*r* = 0.348, *P* = 0.0003). The level of *linc0949* was also reduced in patients with SLE who had the presence of cumulative organ damage. In addition, decreasing expression of *linc0949* was associated with lupus nephritis. *linc0949 *expression significantly increased after treatment, whereas neither disease activity nor organ damage correlated with *linc0597* expression.

**Conclusions:**

Our results provide novel empirical evidence that *linc0949* could be a potential biomarker for diagnosis, disease activity and therapeutic response in SLE.

**Electronic supplementary material:**

The online version of this article (doi:10.1186/s13075-015-0632-3) contains supplementary material, which is available to authorized users.

## Introduction

Systemic lupus erythematosus (SLE) is a systemic autoimmune disease with various clinical manifestations affecting different tissues. It is characterized by the deposition of immune complexes due to widespread loss of immune tolerance to nuclear self-antigens, as well as by excessive proinflammatory cytokine production and damage to multiple organ systems [1]. Recent experimental and clinical studies have placed new emphasis on the role of the innate immune system in SLE. It has become apparent that Toll-like receptors (TLRs) can participate in cell activation by self molecules such as immune complexes containing DNA or RNA. Indeed, TLRs have an important role in the pathogenesis of lupus involving recruitment of adapter proteins; activation of protein kinases and transcription factors; and expression of inflammatory cytokines, chemokines, endothelial adhesion molecules and costimulatory molecules [2]. TLR signaling also stimulates B cell proliferation, cell differentiation and immunoglobulin class switching [2,3].

In the past, the importance of non-protein-coding RNAs has been emphasized in many biological and pathological processes [4]. Much research has been focused on microRNAs (miRNAs). miRNAs are small RNA molecules with a length of approximately 22 nucleotides (nt) that play a critical role in the pathogenesis of SLE by regulating gene expression at posttranscriptional levels [5,6]. miRNAs have also been reported to be involved in the local inflammatory response that ultimately leads to tissue injury and organ damage [7]. Recently, several studies have shown the feasibility of using miRNAs as biomarkers in body fluids for the diagnosis of SLE [8-10]. Though miRNAs play important roles in SLE, they are only a small fraction of the noncoding regions of the mammalian genome. Unlike miRNAs, long noncoding RNAs (lncRNAs) are expressed abundantly, including large intergenic noncoding RNAs (lincRNAs) [11].

lncRNAs are a class of mRNA-like transcripts ranging in length from 200 nt to over 100 kb and lacking any significant open reading frames [12,13]. They are highly diverse and actively present in virtually every aspect of cell biology, such as cell differentiation, cell proliferation, DNA damage response, dosage compensation and chromosomal imprinting. Recently, a number of lncRNA molecules have been reported to be involved in diverse diseases [14-16]. Some evidence indicates that a few samples of lncRNAs could regulate the immune system [17-19]. In particular, there are several emerging hypotheses on lncRNA involvement in rheumatic diseases, such as rheumatoid arthritis (RA) [20,21], autoimmune thyroid disease [22] and psoriasis [23]. Other preliminary data in a murine model system pointed to a link between the lncRNA growth arrest–specific 5 (*GAS5*) and disease susceptibility to SLE [16]. In addition, the chromosomal locus of *GAS5*, 1q25, was showed to be associated with human SLE development in genetic studies [23-25].

Because of the heterogeneous presentation of patients with SLE and their unpredictable disease course, there is a pressing need to identify biomarkers that will facilitate better diagnosis and prognosis, and lincRNAs as biomarkers are still largely unexplored in this regard. It has been reported that four lincRNAs (*linc0949, linc0597, linc1992* and *linc3995*) not only are differentially expressed following innate activation of THP-1 macrophages but also regulate induction of proinflammatory cytokines such as tumor necrosis factor (TNF)-α and interleukin (IL)-6 [26]. Moreover, it is well-established that *IL-6* and *TNF-α* are involved in SLE pathogenesis [27-29].

As mentioned above, we hypothesized that these lincRNAs would produce cross-linking with SLE via innate immunity and play a critical role in the pathogenesis of SLE and that they might serve as biomarkers of disease activity, organ damage and medical response. In the present study, we aimed to investigate whether the expression levels of these lincRNAs in peripheral blood mononuclear cells (PBMCs) were abnormal in patients with SLE, assess the relationship of the levels with disease activity and organ damage, and explore new biomarkers used in disease monitoring and prognostication.

## Methods

### Patients and healthy controls

All samples from patients with SLE and patients with RA were obtained from the Department of Rheumatology of Renji Hospital (Shanghai, China). All patients with SLE met at least four of the American College of Rheumatology (ACR) 1982 revised criteria for SLE [30]. Patients with RA were diagnosed according to the ACR/European League Against Rheumatism 2010 classification criteria for RA [31]. The control group comprised healthy volunteers with no history of autoimmune disease or immunosuppressive therapy. Otherwise eligible individuals with a current or recent infection were excluded from the study. Control subjects were frequency-matched with the patients for age and sex. All participants were from the Han Chinese population. The study was approved by the Research Ethics Board of Renji Hospital, Shanghai Jiao Tong University School of Medicine, Shanghai, China. Informed consent was obtained from all study participants.

The patients with lupus were all receiving steroid therapy at the time of the study, and a prednisone dosage per day (dosages of other steroids were converted to prednisone equivalents) from 2.5 mg to 500 mg (mean dosage: 29.5 mg/day). In addition, 42 patients were receiving immunosuppressive therapy (azathioprine (AZA; n = 7), cyclophosphamide (CYC; n = 10), cyclosporine A (CsA; n = 6), tacrolimus (FK506; n = 1), leflunomide (LEF; n = 2), mycophenolate mofetil (MMF; n = 8), methotrexate (MTX; n = 8)), and 59 were receiving an antimalarial drug (chloroquine or hydrochloroquine). For each patient, the severity of disease was assessed with the Systemic Lupus Erythematosus Disease Activity Index 2000 (SLEDAI-2K) [32]. Organ damage (defined as nonreversible change, not related to active inflammation, occurring since the onset of lupus and present for at least 6 months) was assessed using the Systemic Lupus International Collaborating Clinics/American College of Rheumatology Damage Index (SDI) score [33]. In our cohort, nearly 51.0% of patients (52 of 102 patients with SLE) had either previous or current lupus nephritis (LN) (Table 1). Subjects were considered to have active renal disease if proteinuria was ≥0.5 g/day, hematuria was ≥5 red blood cells per high-power field (hpf), pyuria was ≥5 white blood cells/hpf or cellular casts were present. Infection, kidney stones and other causes were excluded.

**Table 1:** **Large intergenic noncoding RNA** ***linc0949*** **by presence or absence of clinical features of systemic lupus erythematosus** ^**a**^

**Clinical features**		**SLE clinical features present**		**SLE clinical features absent**		***P*** **-value**
		**N**	**Mean ± SEM (range, 10** ^**−3**^ **)**	**N**	**Mean ± SEM (range, 10** ^**−3**^ **)**	
Renal		50	2.16 ± 0.152	52	1.47 ± 0.132	0.0014
Rash		33	1.89 ± 0.200	69	1.87 ± 0.199	NS
Arthritis		35	1.79 ± 0.327	67	1.97 ± 0.163	NS
Serositis		21	1.81 ± 0.307	81	1.72 ± 0.364	NS
Mucosal ulcer		19	2.02 ± 0.207	83	1.87 ± 0.187	NS
Hematologic		30	1.84 ± 0.243	72	2.06 ± 0.179	NS
Neurologic		9	1.99 ± 0.084	93	2.05 ± 0.45	NS
Autoantibodies						
	Anti-dsDNA	40	1.91 ± 0.274	62	1.88 ± 0.264	NS
	Anti-Sm	16	2.07 ± 0.202	86	1.78 ± 0.193	NS
	Anti-nucleosome	40	1.91 ± 0.204	62	1.88 ± 0.212	NS
	Anti-SSA/SSB	32	2.03 ± 0.258	70	2.01 ± 0.176	NS
	Anti-RNP	22	1.95 ± 0.253	80	1.96 ± 0.165	NS
Medical therapy						
	Prednisone dose ≥30 mg/day	48	2.08 ± 0.175	54	1.94 ± 0.181	NS
	Immunosuppressants^b^	42	2.00 ± 0.141	60	1.54 ± 0.151	0.0365

^a^dsDNA, Double-stranded DNA; NS, Not significant; RNP, Ribonucleoprotein; SEM, Standard error of the mean; SLE, Systemic lupus erythematosus; Sm, Smith; SSA, Sjögren’s syndrome–related antigen A; SSB, Sjögren’s syndrome–related antigen B. ^b^Immunosuppressants included azathioprine, cyclophosphamide, cyclosporine A, FK506 (tacrolimus), leflunomide, mycophenolate mofetil and methotrexate.

### Peripheral blood samples handling and RNA processing

Peripheral blood samples (10 ml) were obtained from each subject. The samples were collected in tubes containing ethylenediaminetetraacetic acid (EDTA). PBMCs were isolated from anticoagulated whole blood by use of Ficoll density gradient centrifugation. Then total RNA was extracted from PBMCs using TRIzol reagent (Invitrogen, Carlsbad, CA, USA). The integrity of the RNA was assessed using capillary gel electrophoresis, and the concentrations of RNA were measured using a NanoDrop™ 1000 spectrophotometer (NanoDrop Technologies, Wilmington, DE, USA) with a 260 nm/280 nm ratio above 1.8. About 200 ng of total RNA were reverse-transcribed into cDNA using a PrimeScript RT reagent kit (Takara Bio, Dalian, China). All RNA and cDNA samples were stored at −70°C before use.

### Cell culture and stimulation

Peripheral blood samples were obtained from five healthy donors and five patients with SLE. The samples were collected in tubes containing EDTA. PBMCs were isolated from anticoagulated whole blood by Ficoll density gradient centrifugation. Two hours before stimulation, 1 × 10^6^ PBMCs were cultured in 24-well flat-bottomed plates in 500 μl of RPMI 1640 medium containing 10% fetal bovine serum (FBS). Then the PBMCs were stimulated for 4 hours with the TLR2 ligand Pam3CK4 (20 ng/ml).

### Cell culture and treatment with dexamethasone and immunosuppressant agents

Peripheral blood samples were obtained from two healthy donors. PBMCs were isolated from anticoagulated whole blood by use of Ficoll density gradient centrifugation. PBMCs (1 × 10^6^) were resuspended for 2 hours in 500 μl of RPMI 1640 medium containing 10% FBS, then dexamethasone was added with the indicated concentration (Dexamethasone concentrations were 10ng/ml,100ng/ml,1000ng/ml,10ug/ml, respectively) for another 24 hours, as were CsA (200 nmol) and FK506 (20 nmol). RNA samples were then isolated, and real-time quantitative PCRs (RT-qPCRs) were performed.

### Real-time quantitative polymerase chain reaction

To quantify the expression of four lincRNAs (*linc0949, linc0597, linc1992* and *linc3995*), cDNA was amplified by RT-PCR with SYBR Green (SYBR Premix Ex Taq RT-PCR kit; Takara Bio). The primer sequences used for SYBR Green–based RT-PCR are given in Table 2. The ribosomal protein L13A (*RPL13A*) gene was used as an internal control to normalize the amounts of cDNA. The SYBR Green assays were performed in duplicate using an ABI ViiA 7 Real-Time PCR System (Applied Biosystems, Foster City, CA, USA). The relative expression levels were calculated using the 2^−ΔCt^ comparative threshold cycle method.

**Table 2:** **Primers used to amplify transcripts of large intergenic noncoding RNAs** ^**a**^

**Gene**	**Forward**	**Reverse**
*RPL13A*	5′-CTGGAGGAGAAGAGGAAAGAGA-3′	5′-TTGAGGACCTCTGTGTATTTGTCAA-3′
*ENST00000500597*	5′-TTGGATTCATCCCGTTCACCTCCA-3′	5′-CAGCATGACGATCAAGCGAGATTC-3′
*ENST00000501992*	5′-AACTCCTGACCTCAGGTGATCCAT-3′	5′-AAGGGAGTTTCAGAAGGTGTGGCT-3′
*ENST00000500949*	5′-TCCTGCAACCCAAGGTGGATACTT-3′	5′-CTGCAGTGAGCAGAAATCACGCAT-3′
*ENST00000523995*	5′-GTTTGTGGCATATGGCTCTGCTGT-3′	5′-CATTGCAGGAAAGAGTGCCAAGGT-3′

^a^The four large intergenic noncoding RNAs primers are derived from the literature [26].

### Statistical analysis

Data were analyzed with GraphPad Prism version 5.0 software (GraphPad Software, La Jolla, CA, USA). The nonparametric Mann–Whitney *U* test was used to compare gene expression between two groups. The correlation between groups was evaluated using Spearman’s rank correlation coefficient test. The strength of the correlation was graded using Cohen’s criteria as follows: 0.3 to 0.5 = weak, 0.5 to 0.7 = moderate and >0.7 = strong [34]. *P*-values (two-tailed) <0.05 were considered statistically significant.

## Results

### Decreased *linc0949* and *linc0597* levels in patients with systemic lupus erythematosus

The expression levels of four lincRNAs (*linc0949, linc0597, linc1992* and *linc3995*) in PBMCs taken from 102 patients with SLE, 54 patients with RA and 76 healthy donors were measured using RT-qPCR. Patients with SLE and healthy donors did not differ significantly with respect to mean age or sex distribution (Table 3). The average disease duration of patients enrolled in our study was 4.98 years for those with SLE and 5.12 years for those with RA. In general, the patients with SLE had mild to moderate flares of disease activity and severity, with a mean SLEDAI-2K score of 7 and a mean SDI of 0.78 (Table 3).

**Table 3:** **Demographic data** ^**a**^

	**Patients with SLE (n = 102)**	**Patients with RA (n = 54)**	**Healthy donors (n = 76)**
Age (yr)	34.3 ± 1.3 (16 to 65)	38.7 ± 2.3 (20 to 65)	34.0 ± 1.2 (20 to 63)
Sex (n)			
Female	93	46	70
Male	9	8	6
Disease duration (yr)	4.98 ± 0.76 (0.04 to 28)	5.12 ± 0.84 (0.5 to 20)	–
ANA (%)	95.0	–	–
SLEDAI-2K	6.82 ± 0.52 (1 to 15)	–	–
SDI	0.78 ± 0.14 (0 to 3)	–	–

^a^ANA, antinuclear antibody; RA, Rheumatoid arthritis; SDI, Systemic Lupus International Collaborating Clinics/American College of Rheumatology Damage Index; SLE, Systemic lupus erythematosus; SLEDAI-2K, Systemic Lupus Erythematosus Disease Activity Index 2000 score.

**Figure 1:**
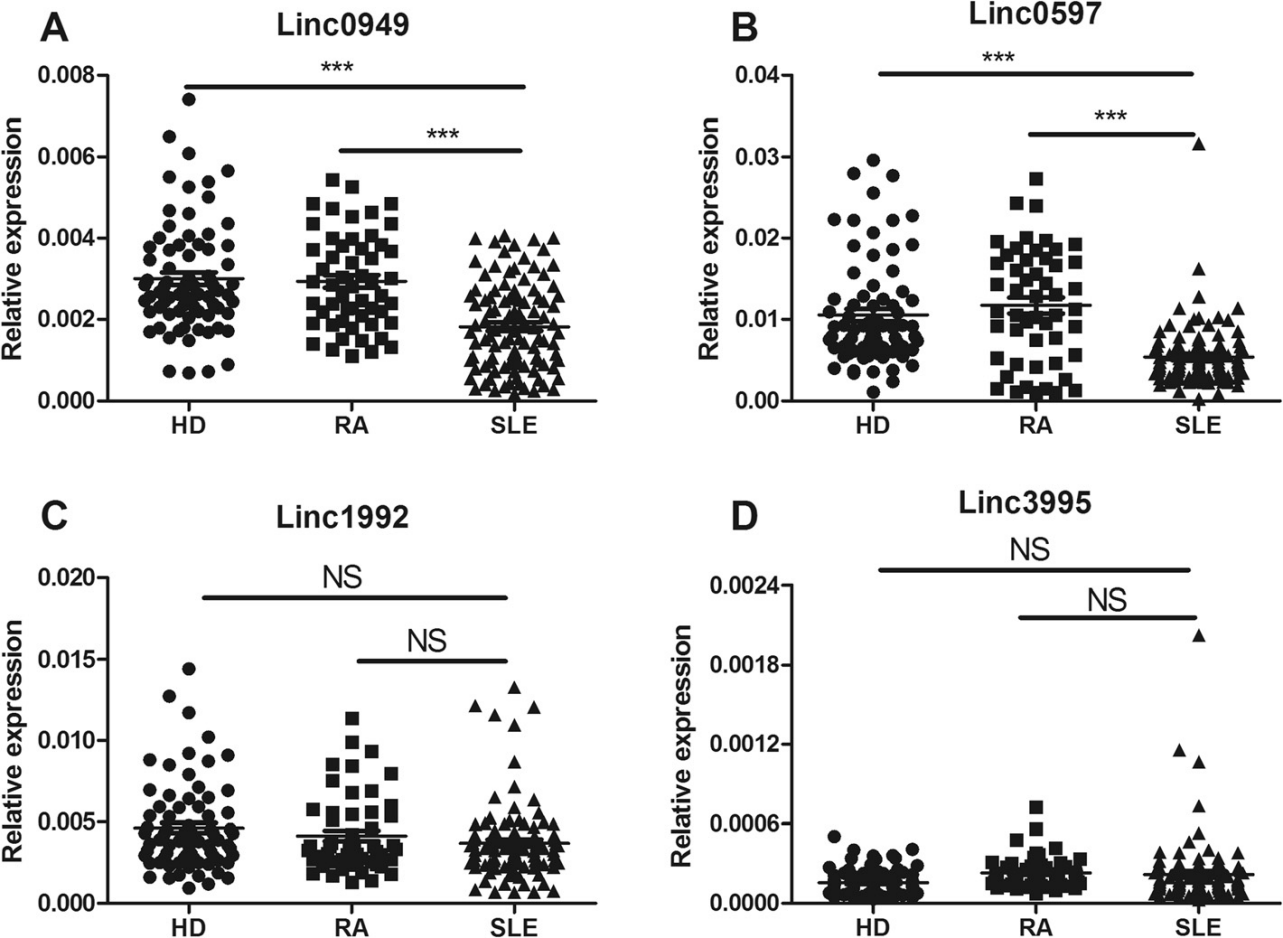
Comparison of expression of four large intergenic noncoding RNAs (*linc0949, linc0597, linc1992* and *linc3995*) between patients with SLE, patients with RA and healthy donors. Each symbol represents an individual patient; horizontal lines indicate median values. The expression levels of the four large intergenic noncoding RNAs (lincRNAs) in 102 patients with SLE, 54 patients with RA and 76 healthy donors were analyzed by real-time quantitative PCR and normalized by RPL13A level. **(A)** Decreased expression of *linc0949* in patients with SLE versus patients with RA and healthy donors. **(B)** *linc0597* expression was significantly reduced in patients with SLE versus patients with RA and healthy donors. No apparent difference was detected in the expression of *linc1992* **(C)** and *linc3995* **(D)** between patients with SLE, patients with RA and healthy donors. NS, Not significant. ***P < 0.001. HD, Healthy donors; RA, Rheumatoid arthritis; SLE, Systemic lupus erythematosus.

As shown in Figure 1A, patients with SLE had significantly lower *linc0949* levels than healthy donors and patients with RA (both *P* < 0.0001). Also, the expression of *linc0597* was decreased dramatically in patients with SLE compared with healthy donors and patients with RA (*P* = 0.0001 and *P* < 0.0001, respectively) (Figure 1B). Figure 1C and Figure 1D, however, show no significant differences in *linc1992* and *linc3995* levels between patients with SLE and healthy donors or patients with RA. These results revealed that lower expression of *linc0949* and *linc0597* was specific to SLE, so we selected these lincRNAs for further research.

### Association of *linc0949* level with disease activity in patients with systemic lupus erythematosus

**Figure 2:**
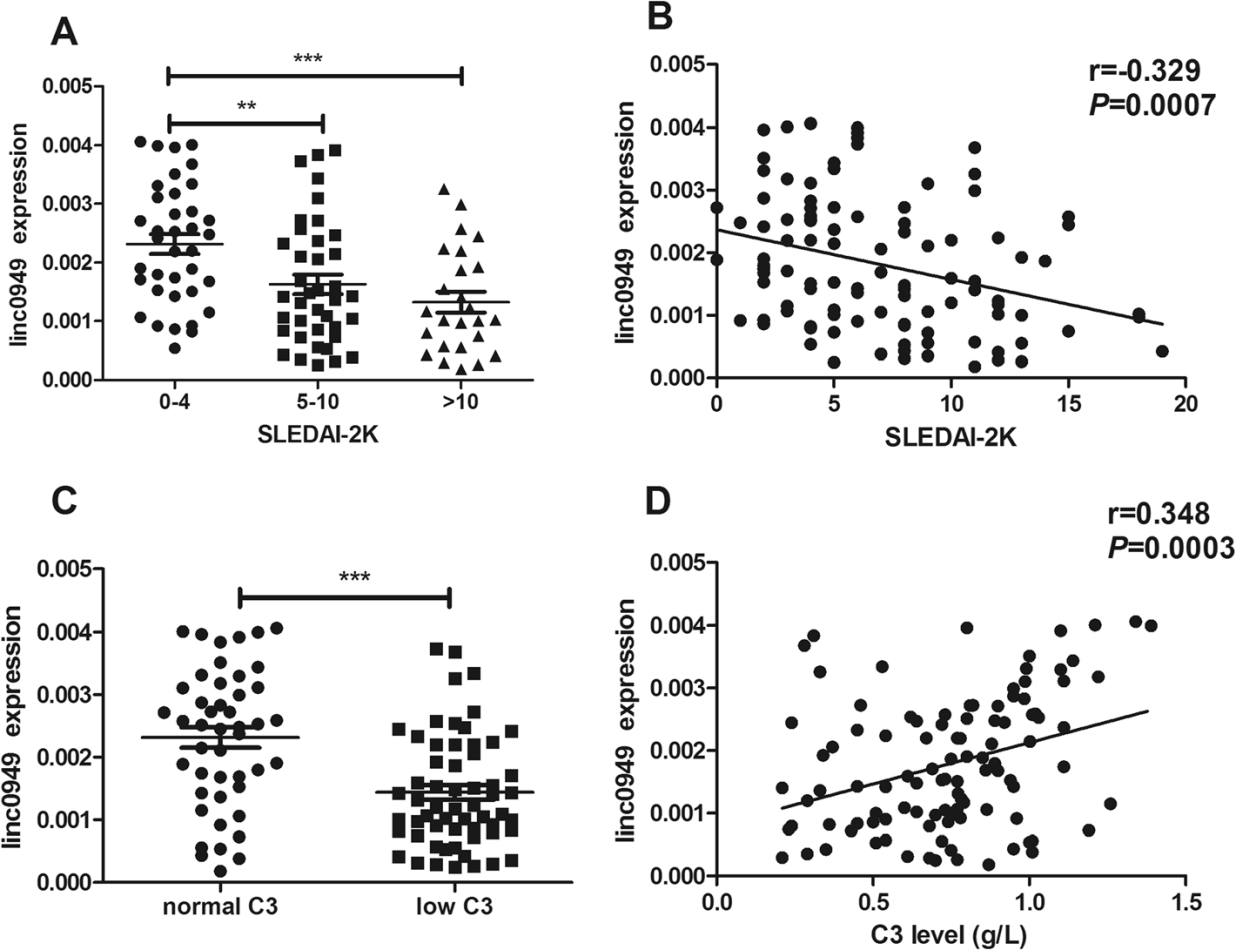
Association of large intergenic noncoding RNA *linc0949* expression with disease activity in patients with systemic lupus erythematosus. **(A)** Patients with systemic lupus erythematosus (SLE) with a moderate to severe flare of disease (Systemic Lupus Erythematosus Disease Activity Index 2000 (SLEDAI-2K) score >10) or a mild flare of disease (SLEDAI-2K score from 5 to 10) had significantly lower large intergenic noncoding RNA *linc0949* expression than did those without a disease flare (SLEDAI-2K score <4) at the time of blood donation. **(B)** *linc0949* expression was negatively correlated with SLEDAI-2K score. **(C)** *linc0949* expression was significantly decreased in patients with SLE with a reduced level of complement C3 (<80 mg/dl) compared with those with normal C3levels. **(D)** A significantly positive correlation was observed between *linc0949* expression and C3 level in patients with SLE. **P < 0.01, ***P < 0.001.

To investigate whether the expression of *linc0949* and *linc0597* is related to SLE disease activity, we compared the relative expression levels of the lincRNAs in patients with SLE with different levels of disease activity, assessed on the basis of SLEDAI-2K score and the level of complement C3. In accordance with the SLEDAI-2K flare system, patients with SLE were divided into those with stable disease (SLEDAI-2K scores from 0 to 4), those with a mild flare (SLEDAI-2K scores from 5 to 10) and those with a moderate to severe disease flare (SLEDAI-2K scores >10). *linc0949* was significantly lower in patients with SLE who had a mild flare or a moderate to severe flare of disease than in patients without a flare (*P* = 0.0032 and *P* = 0.0004, respectively) (Figure 2A). In addition, a correlation between *linc0949* and SLEDAI-2K score was observed in that decreased levels of *linc0949* coincided with increased SLEDAI-2K score in patients with SLE (*r* = −0.329, *P* = 0.0007) (Figure 2B).

C3 level is also an indicator of disease activity. Hypocomplementemia is often observed in patients with SLE with active disease. In the present research, *linc0949* expression was significantly decreased in patients with SLE who had a reduced level of complement C3 (<80 mg/dl) compared with those with normal levels of C3 (*P* < 0.0001) (Figure 2C). Further analysis revealed a positive correlation between *linc0949* level and C3 level (*r* = 0.348, *P* = 0.0003) (Figure 2D). However, the level of *linc0597* did not correlate with SLEDAI-2K and complement C3 level (data not shown). These results indicate that the abnormal expression of *lincRNA0949* may be a key indicator of disease activity in patients with SLE.

### Reduced expression of *linc0949* in patients with systemic lupus erythematosus with organ damage

**Figure 3:**
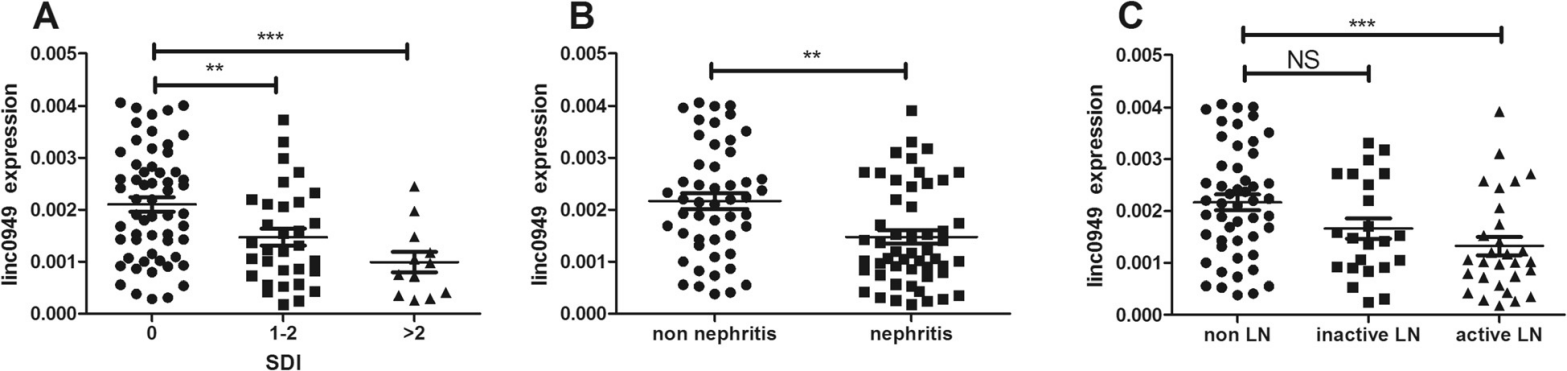
Large intergenic noncoding RNA *linc0949* expression was decreased in patients with systemic lupus erythematosus who had organ damage. **(A)** In our cohort, *linc0949* was dramatically decreased in patients with systemic lupus erythematosus (SLE) who had chronic and irreversible organ damage (Systemic Lupus International Collaborating Clinics/American College of Rheumatology Damage Index (SDI) scores 1 to 2 or more) compared with those with no damage. **(B)** Large intergenic noncoding RNA *linc0949* expression exhibited a decreasing trend in patients with lupus nephritis (LN; n = 52) relative to patients with no history of LN (n = 50). **(C)** Patients with active LN had lower linc0949 expression than those without renal manifestations, whereas *linc0949* level was not significantly different in patients with inactive LN compared with those without LN. NS, Not significant. **P < 0.01, ***P < 0.001.

SLE is a chronic multisystem autoimmune disease that can affect virtually every organ system and may lead to significant morbidities. Assuming that lincRNAs are involved in tissue damage and inflammation, we investigated whether *linc0949* and *linc0597* were associated with different levels of chronic and irreversible organ damage in the patients with SLE. The results revealed significantly lower levels of *linc0949* in patients with SLE with SDI scores of 1 to 2 and in those with scores >2 versus those without organ damage (*P* = 0.0059 and *P* = 0.0009, respectively) (Figure 3A). *linc0597* expression was not significantly reduced in patients with organ damage (SDI ≥1) versus those who remained damage-free (data not shown).

LN is one of the most common clinical manifestations and causes of organ damage in patients with SLE, so we wanted to know whether lincRNA levels are related to LN. In our cohort, nearly 51% of patients (52 of 102 patients with SLE) had either previous or current LN (Table 1). Patients with LN had lower *linc0949* expression levels than those without renal manifestations (*P* = 0.0014) (Figure 3B). Expression levels of *linc0949* were decreased in the group of patients with active LN compared with those without LN (*P* = 0.0009) (Figure 3C), whereas *linc0949* levels were not significantly different in patients with inactive LN compared with patients without LN at the time of blood drawing (*P* = 0.0739) (Figure 3C). These data suggest that *linc0949* expression is related with cumulative organ damage in SLE and that *linc0949* may be useful in predicting long-term outcome and prognosis in patients with SLE.

### Relationship of large intergenic noncoding RNA levels with clinical manifestations and medical therapies

To assess the association between *lincRNA* levels and clinical manifestations, autoantibody profiles and medical treatments, *linc0949* and *linc0597* levels were compared between patients with certain clinical features and those without certain clinical features. We identified no association between *linc0949* or *linc0597* expression and clinical manifestations such as rash, arthritis, serositis, mucosal ulcer, hematologic involvement or neurologic manifestations (*linc0949*: Table 1; *linc0597*: data not shown). We also found that neither *linc0949* nor *linc0597* appeared to be associated with autoantibody production, including anti-double-stranded DNA, anti-Smith antibodies, antinucleosome antibodies, anti–Sjögren’s syndrome–related antigen A and B antibodies and antiribonucleoprotein antibodies (*linc0949*: Table 1; *linc0597*: data not shown).

**Figure 4:**
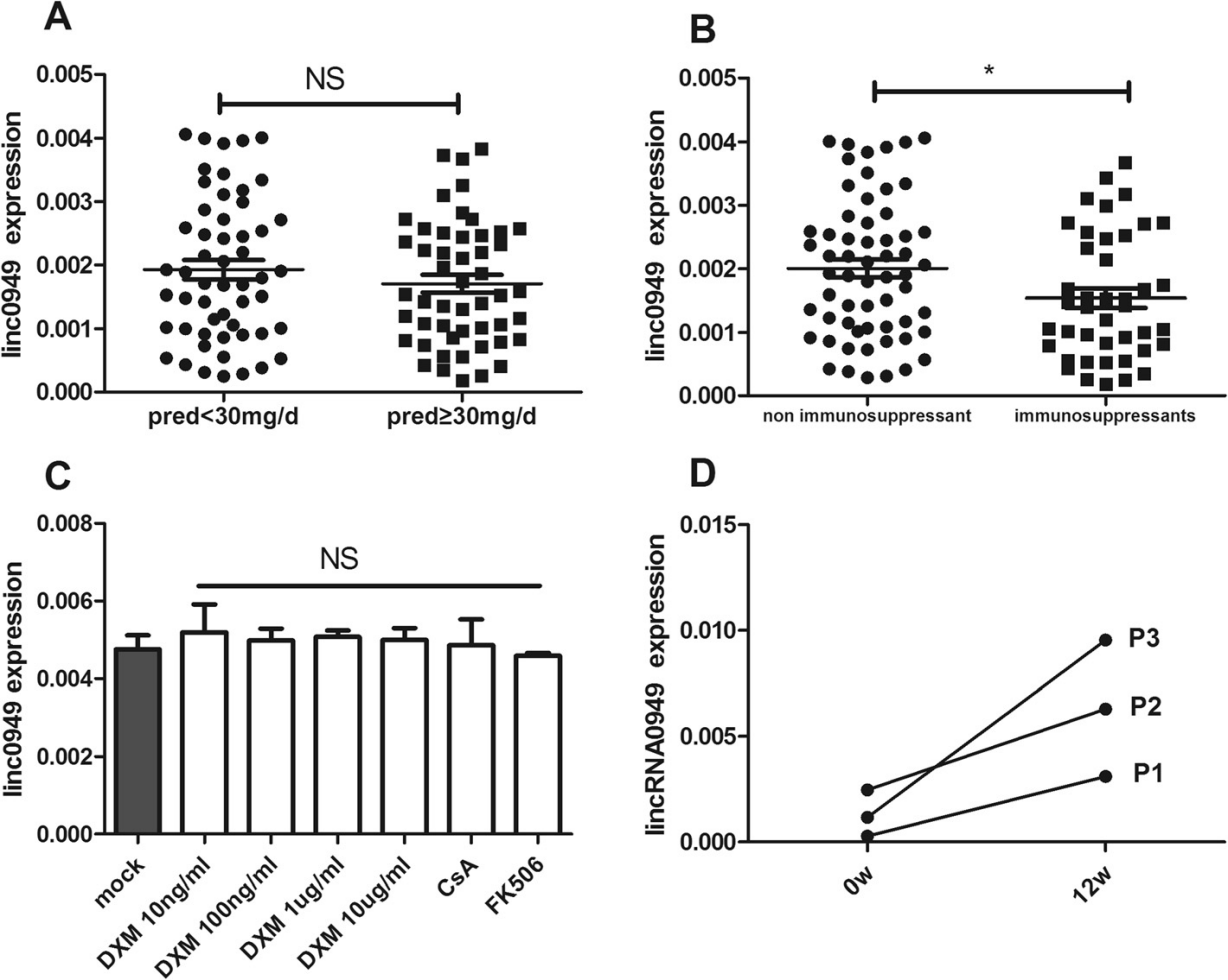
Association of large intergenic noncoding RNA *linc0949* level with medical therapies in patients with systemic lupus erythematosus. **(A)** The level of the large intergenic noncoding RNA *linc0949* had no apparent difference in patients with systemic lupus erythematosus (SLE) whose daily dosage of prednisone was >30 mg compared with those whose daily dosage of prednisone was <30 mg. **(B)** *linc0949* expression decreased significantly in patients with SLE who had used immunosuppressants (azathioprine, cyclophosphamide, cyclosporine A (CsA), leflunomide, mycophenolate mofetil, methotrexate, FK506) compared with those without immunosuppressant treatment. **(C)** The expression of linc0949 was not related to the use of drugs. The data are from two healthy donors. **(D)** *linc0949* expression was detected in three patients with active SLE at the beginning of and after 12 weeks of treatment. As we observed, the *linc0949* expression of all three patients was notably increased when they achieved significant clinical improvement after treatment. *P < 0.05. DXM, Dexamethasone; NS, Not significant; Pred, Prednisone.

When medical therapies were considered, the expression of *linc0949* exhibited no significant difference in patients receiving medium to high doses of prednisone (>30 mg/day) compared with patients treated with low doses of prednisone (Figure 4A). By contrast, the expression of *linc0949* in patients with SLE being treated with immunosuppressants (AZA, CYC, CsA, LEF, MMF, MTX and FK506) was significantly reduced compared with those not receiving immunosuppressants at the time of blood donation (*P* = 0.0365) (Figure 4B).

We next sought to assess whether SLE disease activity or antirheumatic drugs affect the expression of *linc0949*, as it was found that some lncRNAs can be induced in response to the anti-inflammatory agent dexamethasone [35]. Thus, we treated PBMCs of two healthy donors with different concentrations of dexamethasone, CsA and FK506. *FoxP3* level was significantly reduced after treatment (Additional file 1: Figure S1), which supported the fact that dexamethasone and immunosuppressive agents worked effectively *in vitro*. As shown in Figure 4C, dexamethasone, CsA and FK506 did not affect the expression of *linc0949*. This result relates to the effects of antirheumatic drugs on the expression of *linc949*, which confirms that *linc0949* is intrinsically underexpressed in patients with SLE.

We next investigated whether *linc0949* was responsive to treatment and changes over time in conjunction with disease activity. We chose three patients with SLE(P1,P2,P3), as described in Figure 4D, P1 and P3 had initial onset of biopsy-proved type IV LN, P2 had neuropsychological lupus, and their peripheral blood samples were collected at both the beginning of treatment and after 12 weeks of treatment. P1 and P3 used high-dose prednisone (1 mg/kg per day) plus mycophenolate mofetil (1.5-2 g/day), whereas P2 used repeated pulses of glucocorticoid (500 mg intravenous methylprednisolone per day) for three days and then prednisone (1 mg/kg per day) plus monthly pulse of cyclophosphamide (0.8 g/month). After treatment, all of the three patients achieved clinical remission, with the urinary protein level dropping to less than 0.5 g/24 hour in P1 and P3; and with cerebrospinal fluid inspection and head MRI restoring to normal in P2. SLEDAI-2K score of the three patients reduced to a stable level (Additional file 1: Figure S1). In concordance with the clinical improvement, *linc0949* expression in these three patients also significantly increased (Figure 4D).

### Abnormal regulation of lincRNAs during innate activation of peripheral blood mononuclear cells in patients with systemic lupus erythematosus

**Figure 5:**
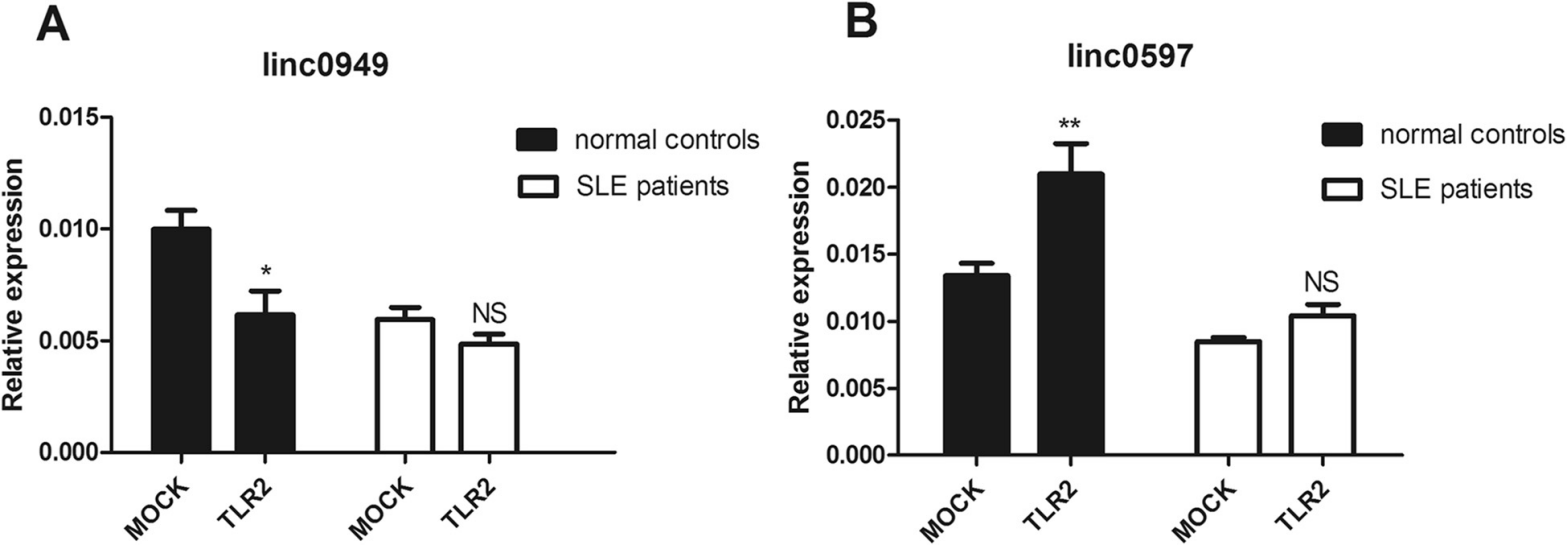
Regulation of large intergenic noncoding RNAs in peripheral blood mononuclear cells by Pam3CSK4. **(A)** Expression of the large intergenic noncoding RNA *linc0949* was lower in untreated and stimulated peripheral blood mononuclear cells (PBMCs) of patients with systemic lupus erythematosus (SLE) than in those of healthy controls. *linc0949* was suppressed by the Toll-like receptor 2 (TLR2) ligand Pam3CK4 (20 ng/ml) in healthy controls, and it was not significantly different after treatment in patients with SLE. **(B)** *linc0597* was upregulated by TLR2 agonist Pam3CK4 (20 ng/ml) in PBMCs of healthy controls, whereas it did not respond to the stimuli in patients with SLE. linc0597 expression was significantly decreased in PBMCs of patients with SLE compared with healthy controls both before stimulation and after treatment. The data shown are from five healthy donors and five patients with SLE after incubation with the indicated stimuli for 4 hours. Real-time quantitative PCR of the indicated RNAs is normalized to RPL13A levels (mean ± standard deviation (SD)). Data are mean ± SD. *P*-values were determined by performing unpaired *t*-tests. NS, Not significant. *P < 0.05; **P < 0.01.

To investigate the different response to innate immunity of *linc0949* and *linc0597* in healthy donors and patients with SLE, we stimulated PBMCs from healthy donors and patients with SLE with TLR2 ligands. We chose TLR2 ligands because it was reported that *linc0949* and *linc0597* were regulated in THP-1 macrophages following Pam3CSK4 stimulation [26] and TLR2 was required for the production of prototypical lupus autoantibodies and the development of renal disease in murine lupus [36,37]. To prepare cell samples, PBMCs obtained from 5 healthy donors and 5 patients with SLE were stimulated with Pam3CSK4 for 4 hours. Then we performed RT-qPCR to identify changes in lincRNA expression. In PBMCs of healthy controls, *linc0949* was suppressed (Figure 5A), while *linc0597* was increased (Figure 5B) after treatment with Pam3CSK4. But in patients with SLE, *linc0949* and *linc0597* could not response to the stimuli compared with healthy donors (Figure 5A and B). These results demonstrated that lincRNAs were indeed involved in the complex regulatory network of innate immunity.

## Discussion

In recent years, an increasing body of evidence has shown that lncRNAs play major biological roles in embryogenesis, stem cell biology and cellular development and show developmental and tissue-specific expression patterns [11,38,39]. Studies have also suggested that abnormal expression of lncRNAs might be associated with numerous diseases, indicating that these RNAs may open a new avenue for diagnostic and therapeutic targets by recognition of their roles in human disease.

In the present study, we detected four lincRNAs (*linc0949*, *linc0597*, *linc1992* and *linc3995*) and investigated the association between their expression levels and specific clinical features of SLE. Two of these lincRNAs (*linc0949* and *linc0597*) were significantly decreased in patients with SLE compared with healthy donors and disease controls. *linc0949* was associated with disease activity, as assessed using the SLEDAI-2K score and C3 level in patients with SLE. Moreover, *linc0949* expression was reduced in patients with SLE with ongoing or cumulative organ damage, as assessed based on SDI score or the presence of active LN. *linc0949* expression does not participate in clinical manifestations other than LN, which demonstrates that it has very good detection specificity for LN. Lower levels of *linc0949* may thus be helpful to identify patients with SLE who have active and severe disease. To evaluate the effect of antirheumatic drugs on the expression of *linc949*, we used *in vitro* studies to test whether the addition of dexamethasone, CsA or FK506 to cultured PBMCs would affect the expression of *linc0949*. As shown in Figure 4C, these antirheumatic drugs did not affect the expression of *linc0949* in PBMCs, which confirmed that *linc0949* was intrinsically underexpressed in patients with SLE. *linc0949* expression of three patients with severe disease flares significantly increased after treatment (Figure 4D), indicating that *linc0949* might be responsive to treatment and might change in conjunction with disease activity and severity and suggesting that *linc0949* might be used to monitor disease progression and guide therapy.

Over the past several decades, tremendous enthusiasm and efforts have been devoted to biomarkers for SLE because the diagnosis of SLE requires a combination of clinical manifestations and biomarkers and no single test is sufficiently sensitive and specific to be diagnostic. The traditional antibodies fail to identify the pathogenic processes, organ damage and biological responses to a therapeutic intervention. Many groups, including the members of our laboratory, have found a set of potential biomarkers for SLE. For example, interferon (IFN)-induced genes and IFN-inducible chemokines may serve as new biomarkers for active and severe disease in patients with SLE [40,41]. Some limitations of these biomarkers are revealed gradually, however. Several studies have shown that overexpressed transcripts of the type I IFN pathway are also identified in patients with myositis, RA, Sjögren’s syndrome and scleroderma [42-44], so an IFN signature or chemokine is not sufficiently specific. In two longitudinal studies to date, researchers have reported conflicting results on the correlations between type I IFN gene signature score and diseases activity [45,46]. There is an urgent need for SLE biomarkers that can help enhance comprehension of the mechanisms of diseases or effects of therapies by relating the changes of molecular and cellular pathways to disease status or clinical responses. In our present study, we demonstrate that lower expression of *linc0949* is specific for SLE and that it is helpful in identifying disease activity, monitoring disease progression and guiding therapy. However, *linc0949* needs to be further investigated in large-scale multicenter trials.

On the basis of our present observations, we believe that *linc0949* could be a potentially readily accessible biomarker useful for diagnosing SLE. As a novel biomarker, lincRNAs have the following characteristics. First, lincRNAs display a wide range of stabilities in the samples comparable to those of mRNAs of protein-coding genes [47]. Second, they show a greater tissue specificity compared with protein-coding mRNAs and miRNAs, which are frequently expressed in multiple tissues, and they show highly increased or decreased expression levels in disease [15]. In addition, lincRNAs are also detectible in body fluids such as plasma and urine [48-50], diagnostic samples of which are easy to collect using noninvasive methods. Moreover, detection of the lincRNAs is simple, inexpensive and has high throughput, making it a suitable approach to gaining an overview of disease activity and severity in patients with SLE. These features make lincRNAs very suitable as biomarkers, and many studies have been published on this matter in recent years, both in cancer and in other human diseases such as cardiovascular diseases [50] and neurological disorders [51].

Most lncRNAs described to date have been found to be related to transcriptional regulation or mRNA processing, characteristics that they share with microRNAs. However, unlike microRNAs, lncRNAs show a greater complexity of their functions and have a wider spectrum of biological contexts, such as epigenetic regulation, enhancer-like function and RNA splicing, editing and export [52]. In our ongoing experiments, we found that *linc0949* and *linc0597* could be induced by TLR2 in PBMCs of healthy donors, but they did not respond to the stimuli in patients with SLE as compared with healthy donors (Figure 5). These results validate that lincRNAs were indeed involved in the complex regulatory network of innate immunity. We hypothesized that the regulation defect of *linc0949* and *linc0597* could contribute to the pathogenesis of SLE and that lincRNAs may provide potential novel strategies for therapeutic intervention, although their function and mechanism of action need further exploration.

We have suggested the abnormal expression of *linc0949* in patients with SLE, as well as the association of lincRNA level with disease activity and organ damage; however, in this study, we did not conduct a functional study of this lincRNA, and the underlying mechanism needs further investigation. We did not detect the expression of *linc0949* released in the local target tissues and in specific cell subsets in PBMCs. Future studies are needed to investigate the lincRNA expression level in specific organ and cell types as well.

## Conclusions

We found that the expression of two lincRNAs was dramatically reduced in patients with SLE and that the decreasing level of *linc0949* was correlated with disease activity, degree of organ damage and medical therapies in patients with SLE. *linc0949* may serve as a potential biomarker for diagnosis, disease activity and therapeutic interventions in patients with SLE.

## Additional file

Additional file 1: Figure S1. Effectiveness of the amplified drugs and SLEDAI score flare of three patients.

## Abbreviations

ACR: American College of Rheumatology
ANA: Antinuclear antibody
AZA: Azathioprine
C3: Complement component 3
CsA: Cyclosporine A
CYC: Cyclophosphamide
dsDNA: Double-stranded DNA
DXM: Dexamethasone
EDTA: Ethylenediaminetetraacetic acid
FBS: Fetal bovine serum
FK506: Tacrolimus
*GAS5*: Growth arrest–specific 5
hpf: High-power field
IFN: Interferon
IL: Interleukin
LEF: Leflunomide
*linc0597*: *ENST00000500597*
*linc0949*: *ENST00000500949*
*linc1992*: *ENST00000501992*
*linc3995*: *ENST00000523995*
lincRNA: Large intergenic noncoding RNA
LN: Lupus nephritis
lncRNA: Long noncoding RNA
miRNA: microRNA
MMF: Mycophenolate mofetil
MTX: Methotrexate
NS: Not significant
nt: Nucleotide
PBMC: Peripheral blood mononuclear cell
RA: Rheumatoid arthritis
RNP: Ribonucleoprotein
*RPL13A*: Ribosomal protein L13A gene
RT-qPCR: Real-time quantitative polymerase chain reaction
SDI: Systemic Lupus International Collaborating Clinics/American College of Rheumatology Damage Index
SEM: Standard error of the mean
SLE: Systemic lupus erythematosus
SLEDAI-2K: Systemic Lupus Erythematosus Disease Activity Index 2000
SLICC: Systemic Lupus International Collaborating Clinics
Sm: Smith
SSA: Sjögren’s syndrome–related antigen A
SSB: Sjögren’s syndrome–related antigen B
TLR: Toll-like receptor
TNF: Tumor necrosis factor
SEM: Standard error of the mean
