# Supplementary material for: Association of large intergenic noncoding RNA expression with disease activity and organ damage in systemic lupus erythematosus
Source: Arthritis Res Ther. 2015 May 21;17(1):131. doi: 10.1186/s13075-015-0632-3 (PMC4440330; doi:10.1186/s13075-015-0632-3)
Supplement: Additional file 1: Figure S1. — Effectiveness of the amplified drugs and SLEDAI score flare of three patients. [file 13075_2015_632_MOESM1_ESM.pdf]

Figure S1

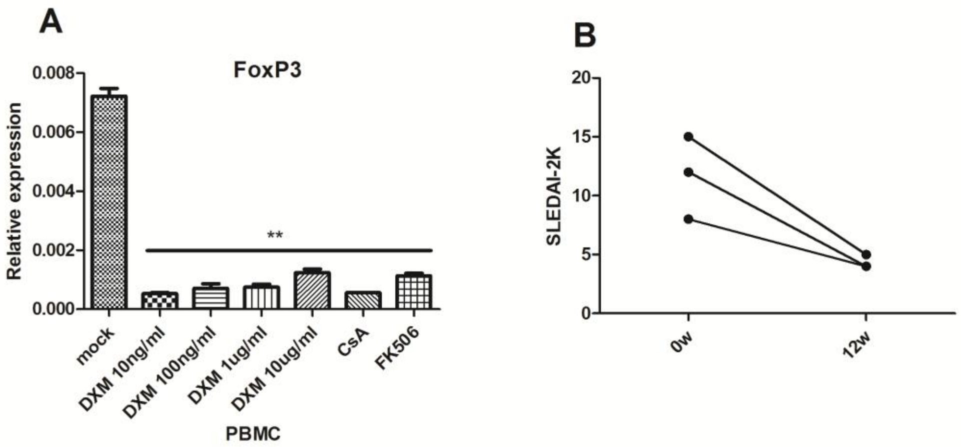

Figure S1 Effectiveness of the amplified drugs and SLEDAI score flare of three patients (A) FoxP3 levels significantly reduce after treatment. Results above presented as mean ±SD; ns, no significance,* *, *p*<0.01. Results above were from two healthy donors. The expressions of FoxP3 were analyzed by RT-qPCR and normalized by RPL13A level. **(B)** SLEDAI score of three patients flare to 4-5 after 12 weeks of treatment.
